# Optimization of In Vitro Ovule Culture System in Upland Cotton

**DOI:** 10.3390/plants14182936

**Published:** 2025-09-22

**Authors:** Li Zhang, Congcong Zheng, Aijuan Wang, Xuehui Huo, Xiaoying Wu, Jialin Liu, Yupeng Fan, Jianlong Dai, Fanchang Zeng

**Affiliations:** 1 College of Agronomy, Shandong Agricultural University, Tai’an 271018, China; zhangli123sdau@163.com (L.Z.); zccylxq@163.com (C.Z.); 13001755317@163.com (A.W.); xuehui@sdau.edu.cn (X.H.); 18369503515@163.com (X.W.); ljl1005819@126.com (J.L.); 2 College of Life Sciences, Huaibei Normal University, Huaibei 235000, China; 3 Institute of Industrial Crops, Shandong Academy of Agricultural Sciences, Jinan 250100, China

**Keywords:** cotton ovules, in vitro culture, carbon sources, kinetin, glutamine

## Abstract

In vitro ovule culture serves as an experimental platform for exploring the growth and development processes of cotton fibers. However, over the decades, research on the in vitro ovule culture of upland cotton has remained underdeveloped. In this study, ovules collected 2 days post-anthesis (2 DPA) from the upland cotton genetic standard line TM-1 were used to investigate the effects of carbon sources (glucose, fructose, sucrose), kinetin (KT), and glutamine (Gln) on ovule growth and observed fiber development in vitro. The results showed that the ovules grew more favorably on a medium supplemented with 0.05 M glucose, 0.02 M fructose, and their degradation products as carbon sources. Regarding the role of KT, it has a slight inhibitory effect on the development of cotton fiber in vitro at a lower concentration (0.1 mg/L). However, as the concentration increased (0.5 mg/L), its effect shifted to promotion. Additionally, Gln demonstrated the ability to enhance the characteristics of fiber fluffiness. In this study, the optimized formula for the in vitro ovule culture of upland cotton was established. This method provides an improved technical system for the in vitro ovule culture of upland cotton, holding great potential for fiber function genomics and seed bioengineering in cotton.

## 1. Introduction

Cotton (*Gossypium hirsutum* L.) is a vital fiber crop and a key raw material for the textile industry, playing a significant role in the realm of textile fibers [1]. With the continuous development of the economy, the textile industry’s demand for high-quality cotton fibers is on the rise. Cotton fiber, a highly elongated cell derived from the ovule epidermis, is a characteristic feature of the genus *Gossypium* [2,3]. Studies have discovered that in vitro fiber cell growth and development closely resemble the natural situation [4,5]. Consequently, cotton fiber development can be studied through the in vitro culture of cotton ovules [6]. The induction of cotton fiber cell growth via an in vitro cotton ovule culture system provides a valuable and practical platform for studying the effects of plant growth regulators and other chemicals on cotton fiber cell development in vitro [7,8]. This approach enables the conduct of physiological and biochemical tests that are not easy to implement in the field.

Previous research revealed that the most substantial fiber growth occurred in ovules collected one day after anthesis [9]. Subsequently, the researchers developed a medium (BT medium) suitable for the in vitro culture of cotton ovules [10,11]. Since then, the majority of related studies have been based on this medium. As more and more relevant research has been carried out, the in vitro culture of ovules has gradually become an essential means of studying fiber development. Researchers cultured cotton ovules in vitro under both light and dark conditions and found that the expression levels of fiber elongation genes and fiber length were significantly lower under light conditions than under dark conditions [12]. It has been reported that brassinosteroids and peroxidase play a crucial role in the elongation of cotton fibers [13,14,15]. Auxins and gibberellins are also well known to play important roles in fiber growth [16,17,18,19,20,21]. Momtaz found that KT alone could not promote fiber growth [22]. There was a study on the cultivation of microstem tips from aseptic upland cotton seedlings. Ganesan and Jayabalan studied the impact of treating glucose and other carbon sources on the medium value of cotton ovules [23]. The results showed that among carbon sources, for disaccharides, waste sugar was a viable option, and for monosaccharides, glucose was more effectively utilized.

In rapidly developing plant tissues such as cotton ovules and their fibers, nutrient availability, especially nitrogen, is paramount. While inorganic nitrogen sources are typically provided in culture media, organic forms like glutamine (Gln) often play a crucial role in enhancing growth and development [24,25]. Glutamine is a central amino acid in plant metabolism, vital for efficient nitrogen assimilation and transport. It serves as a direct precursor for protein synthesis, a process that is highly active during intense cell division and expansion, characteristic of fiber elongation [26]. Furthermore, glutamine contributes to maintaining the carbon–nitrogen balance within plant cells and can even aid in osmotic adjustment, which is particularly relevant in the controlled yet sometimes stressful in vitro culture environment [27,28]. Given the high metabolic demands of developing cotton ovules and their fibers, we hypothesized that supplementing the culture medium with glutamine would provide a readily available and efficiently utilized organic nitrogen source, thereby further supporting and enhancing ovule growth and fiber development beyond what inorganic nitrogen sources alone might achieve.

Building on previous work on cotton ovule culture, which has individually explored components like carbon sources, plant hormones, and nitrogen sources [29,30,31], our study distinguishes itself through a comprehensive and integrated optimization strategy. We simultaneously investigate the synergistic effects of three crucial medium components: specific ratios of glucose and fructose and their associated degradation products, kinetin levels, and glutamine. Unlike previous research that often focused on optimizing one or two factors in isolation, our approach aims to identify a precise and effective combination that supports ovule growth and observed fiber development. Specifically, our work provides insights into the combined action of a tailored carbon source blend with distinct concentrations of kinetin and glutamine, identifying a mix that qualitatively promotes favorable ovule and fiber growth. This integrated perspective and the identification of such specific effective combinations for upland cotton represent a novel contribution, offering directly applicable information to refine in vitro culture systems for this important crop.

Over the decades, research on the in vitro culture of upland cotton ovules has remained relatively limited and underdeveloped, leading to progress stagnation in this field. The present study aims to explore the effects of key culture components, such as carbon sources, KT, and Gln, on the growth and development of cotton ovules under in vitro conditions. Based on these findings, we optimized the medium formula, which enables the ovules to develop more stably and effectively in vitro. This research is expected to contribute to the establishment of a reliable in vitro ovule culture system in cotton, which will provide an important experimental platform for fiber function genomics and seed bioengineering.

## 2. Results

### 2.1. Effect of Carbon Sources on In Vitro Culture of Cotton Ovules

**Figure 1:**
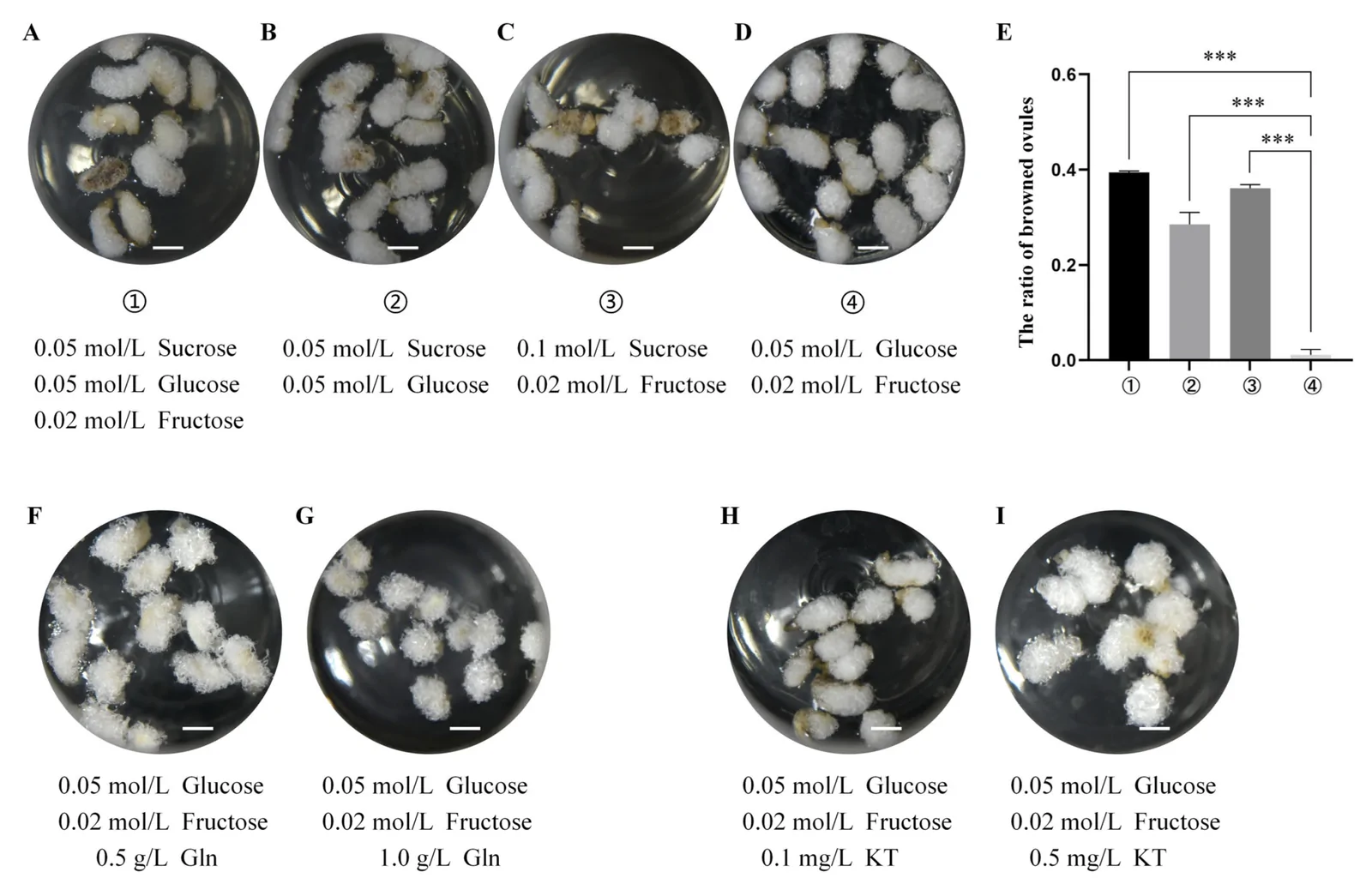
In vitro culture of cotton ovules under different medium conditions. (**A**–**E**) Effect of carbon sources on cotton ovule culture in vitro. Tukey’s test was used for statistical analysis. Error bars indicate standard error of the mean (SEM), with statistical significance indicted by *** *p* < 0.001. (**F**,**G**) Effect of glutamine (Gln) on cotton ovule culture in vitro. (**H**,**I**) Effect of kinetin (KT) on cotton ovule culture in vitro. Scale bars represent 1 cm.

In this study, we selected three carbon sources, glucose, sucrose, and fructose. We set up four different carbon source combinations and proportions to study the effects of carbon sources on ovule growth and fiber morphology during the in vitro culture of cotton ovules. The developmental status of the structures outside the fibers after culturing the ovules in vitro for 24 days is shown in Figure 1A–E.

When the carbon source of the medium was 0.05 M glucose + 0.02 M fructose, the fiber growth and development of the ovules in vitro were in good condition, with many and dense fibers and no browning phenomenon. For the other three carbon source combinations, the ovules had different degrees of browning (Figure 1A–E). Therefore, it was determined that the most suitable carbon sources for the in vitro culture of upland cotton ovules were glucose and fructose.

### 2.2. Effect of Gln on In Vitro Culture of Cotton Ovules

Using glucose and fructose as carbon sources, the effects of Gln (0.5 g/L and 1.0 g/L) on ovule culture in vitro were investigated (Figure 1F,G). The group without Gln was the control group (Figure 1D). The fibers of the ovules in the group supplemented with 0.5 g/L of Gln were significantly fuller than those in the 1.0 g/L of Gln group (Figure 1F,G), while the fibers of the ovules cultured in vitro in the control group were relatively tight and dense (Figure 1D). The results indicated that Gln could, to a certain extent, enhance the qualitatively observed fluffiness of cotton ovule fibers.

### 2.3. Effect of KT on In Vitro Culture of Cotton Ovules

**Figure 2:**
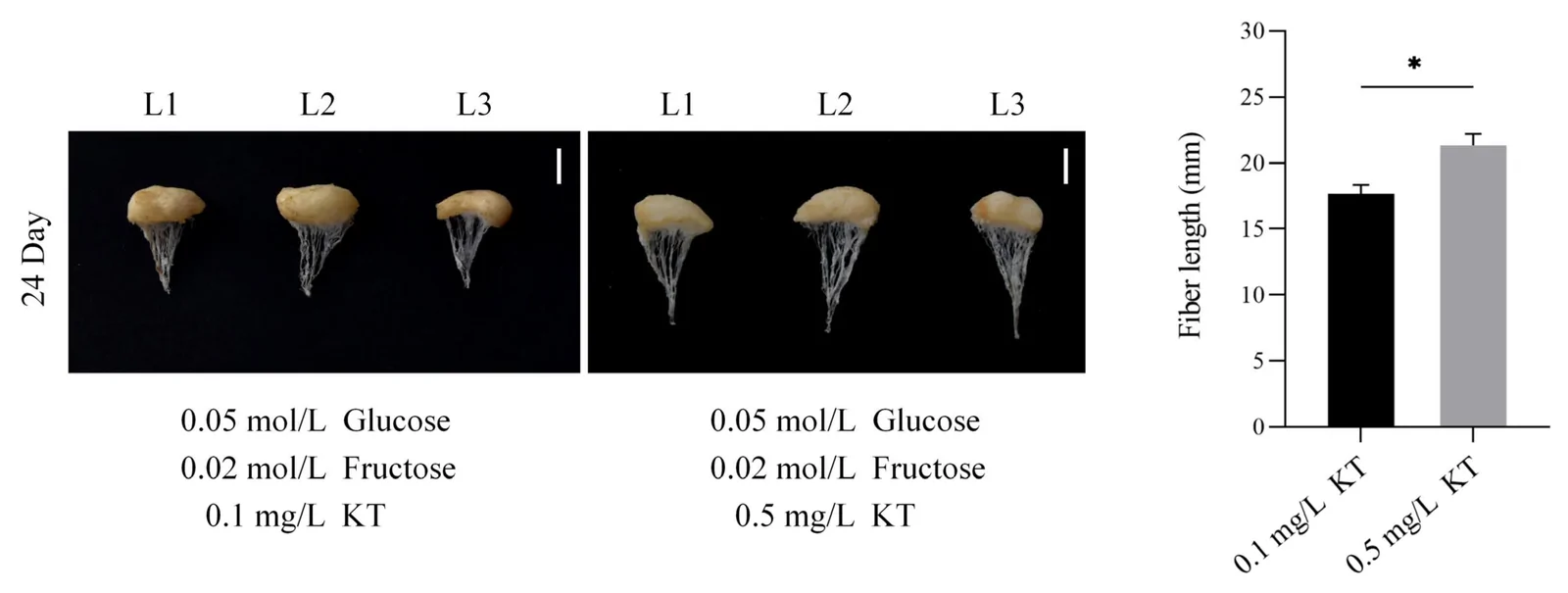
The phenotypic characteristics and length of the fibers under different KT concentrations after 24 days of cultivation. The experiment was performed using three different cotton lines (L1, L2, and L3) to confirm the reproducibility of the results. * *p* < 0.05. Scale bars represent 7 mm.

The above results found that the ovules of upland cotton grew more readily on the medium with glucose and fructose as carbon sources, so we used these two sugars as carbon sources in the subsequent experiment. We studied the effects of KT at concentrations of 0.1 mg/L and 0.5 mg/L on the in vitro culture of ovules (Figure 1H,I). We used media without KT as a control group (Figure 1D). Compared with the control group, the addition of a low concentration of KT had an inhibitory effect on fiber growth. With the increase in KT concentration, the inhibitory effect was weakened. Specifically, the fiber length of ovules cultured with 0.5 mg/L of KT was longer than that with 0.1 mg/L of KT (Figure 2). Moreover, the fibers were qualitatively observed to have a high abundance and a loose, voluminous appearance, presenting a more desirable appearance than the control. In subsequent experiments, a KT concentration of 0.5 mg/L was selected to optimize the formulation.

### 2.4. Observation of Fiber Changes During In Vitro Culture of Ovules on Optimized Medium

**Figure 3:**
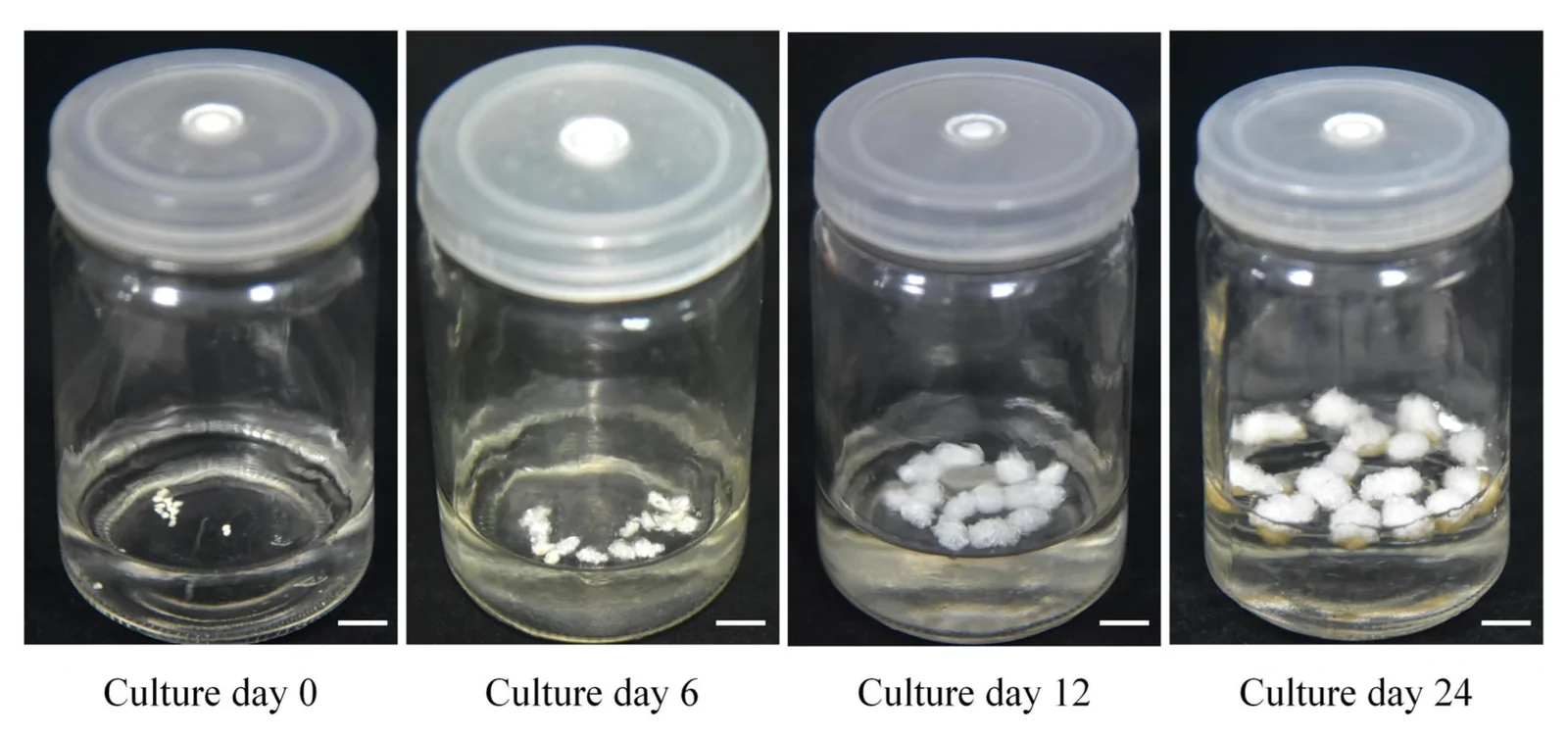
Growth process of the cotton ovule in vitro culture. Scale bars represent 1 cm.

To some extent, the ovule culture system was optimized in this study (Table 1). In this study, we observed the developmental changes of ovules that were grown on a medium containing glucose and fructose as carbon sources with the addition of 0.5 mg/L of KT and 0.5 g/L of Gln. As can be seen from Figure 3, the fiber development of the ovule in vitro culture changed significantly, and the fibers were well-developed and showed no morphological abnormalities.

## 3. Discussion

Cotton ovule culture is an important method for studying the growth and development of cotton fibers and improving their yield and quality. Building on the previous findings presented, our study’s significance extends to providing a more integrated understanding of cotton ovule culture. By systematically optimizing the interplay between carbon sources, kinetin, and glutamine, we have identified specific medium conditions that qualitatively promote favorable ovule growth and fiber development in upland cotton. Our multi-parametrical examination offers valuable insights beyond single-factor analyses by systematically identifying the combined effect of crucial medium components. This approach contributes to the broader field of cotton fiber biology and enhancing the foundational knowledge necessary for advanced in vitro cultivation and biotechnological applications. It provides a technical platform for studying cotton fiber development.

The selection of the medium carbon source is very important, as different carbon sources have different effects on fiber growth and development. This study has shown that sucrose and glucose are relatively ideal carbon sources and have little effect on the pH of the medium [23]. Beasley found that fructose had little effect on fiber growth and development but reduced browning [10,11]. The results of this study showed that glucose and fructose were the most suitable carbon sources for upland cotton ovule culture systems, which is consistent with previous studies. Our findings indicate that when the culture medium contained 0.05 M glucose and 0.02 M fructose, the ovules not only exhibited robust fiber growth but also notably lacked any browning. In contrast, the other carbon source combinations led to varying degrees of browning. This difference can be largely attributed to the delicate balance between carbon supply and osmotic potential within the in vitro culture environment. High concentrations of sugars, while providing essential carbon, can significantly increase the osmotic potential of the culture medium. When the external osmotic potential is too high, it can induce osmotic stress in the explants, impairing their ability to absorb water and nutrients efficiently [32,33]. This stress often triggers a plant’s defense mechanisms, including the increased production and subsequent oxidation of phenolic compounds, which polymerize into dark, insoluble pigments causing the characteristic browning [34,35]. The absence of browning at 0.05 M glucose + 0.02 M fructose suggests that this specific combination of sugars and their degradation products provides an optimal balance, possibly by allowing degradation products such as lactic or oxalic acid to significantly downregulate secondary metabolism. This offers sufficient readily available carbon for vigorous metabolic activity and growth yet without imposing the excessive osmotic stress that would otherwise lead to a physiological imbalance and the detrimental browning response. Conversely, the browning observed in other combinations might stem from either suboptimal sugar levels, leading to carbon starvation stress, or excessively high total sugar concentrations, creating undue osmotic pressure, both of which can compromise cellular integrity and trigger oxidative processes. This highlights the critical importance of precisely optimizing carbon source concentrations not only for nutrient supply but also for maintaining a favorable osmotic environment, essential for explant health and successful in vitro culture.

An early study found that the addition of KT promoted the in vitro culture of ovules, and another study showed that when 1.0 mg/L of NAA was combined with 1.0 mg/L of KT, the length of cotton fibers reached the maximum value [4,36]. In this study, it was found that certain concentrations of KT could promote the development of ovules in vitro. However, only two concentrations of KT were studied in this experiment. Compared with 0.1 mg/L of KT, the fiber growth was better when 0.5 mg/L of KT was added. This aspect can be further researched in the future.

In this study, fiber fluffiness using the Gln-supplemented medium was qualitatively observed to be higher than that of the control medium. The increase in fluffiness is conducive to the separation of fibers from the ovule so as to characterize fiber properties more conveniently. This qualitative observation suggests that Gln contributes to more desirable fiber properties, potentially enhancing characteristics like hygroscopicity and air permeability. From a physiological perspective, glutamine is a crucial organic nitrogen source and a central hub in plant nitrogen metabolism. It is directly involved in protein synthesis, a process fundamental to cell division and expansion, which are critical for fiber elongation and development [26,37]. The increased perceived fluffiness could indicate more robust fiber elongation or a different arrangement of cellulose microfibrils influenced by improved protein synthesis or turgor maintenance. Furthermore, glutamine plays a role in maintaining the carbon–nitrogen balance and can contribute to osmotic adjustment within plant cells, which could create a more favorable intracellular environment for fiber development in culture [27,28]. While our current observations are qualitative, they align with broader plant tissue culture research that often shows the beneficial effects of organic nitrogen sources, like glutamine, in promoting vigorous growth and differentiation, often by bypassing limitations associated with solely inorganic nitrogen or by directly supporting key metabolic pathways. These findings suggest that glutamine is a valuable component for optimizing cotton ovule culture for fiber development. Overall, we have successfully optimized a medium that consistently and visibly promotes favorable ovule growth and enhanced fiber development. Our study provides a reliable protocol based on clear qualitative observations, and we believe it lays a strong and valuable foundation for future quantitative research. This reproducible method serves as a valuable tool for the scientific community, allowing for more detailed investigation into the roles of specific components like glutamine.

In the field of plant tissue culture, the sterilization of media is a critical step in ensuring sterility, but it is important to acknowledge that this process can alter the chemical composition of the medium. As recognized in a study, carbohydrates like glucose and fructose are susceptible to degradation under the high heat and pressure of autoclaving [38]. This phenomenon is a well-known consideration in pharmaceutical and biological research, where the formation of glucose degradation products (GDPs) can occur through oxidation, hydrolysis, and dehydration reactions. The formation of these products, which can also cause a decrease in the medium’s pH, may influence the physiological responses of plant tissues. While our study began with the preparation of media using purified glucose and fructose, the final medium, following autoclaving, is likely a complex solution containing a mixture of the intended carbon sources and their degradation byproducts. Therefore, the observed effects on ovule culture are likely due to the combined action of both the original carbohydrates and the resulting GDPs. Acknowledging this, our findings reflect the overall effect of these carbon sources under standard tissue culture conditions, offering valuable practical insights for the optimization of cotton ovule culture. Future studies could employ alternative sterilization methods, such as filter sterilization, or incorporate sterile-filtered sugars after autoclaving to more precisely isolate the effects of individual carbohydrates. Our current work, however, provides a robust evaluation of media as it is commonly prepared and used in the laboratory.

## 4. Materials and Methods

### 4.1. Plant Materials and Planting Conditions

The cotton variety genetic standard line TM-1 (*Gossypium hirsutum* L.), ordered from and stocked in the germplasm center of the Institute of Cotton Research in the Chinese Academy of Agricultural Sciences (CAAS), was planted in the experimental field of Shandong Agricultural University (Tai’an, Shandong, China). The upland cotton genetic standard line TM-1 is widely used as a parent in upland breeding. Before sowing, during land preparation, base fertilizer was applied, primarily consisting of organic fertilizer combined with a low-nitrogen, high-phosphorus, high-potassium compound fertilizer and trace element fertilizers. This provided a nutritional foundation for the cotton’s entire growth period. During the growing season, additional compound fertilizers were top-dressed according to the needs of different growth stages: a balanced compound fertilizer was applied during the squaring stage, and a high-nitrogen, high-potassium compound fertilizer was applied during the flowering and boll-forming stages. Application rates were adjusted based on soil fertility, plant vigor, and climatic conditions to ensure scientific fertilization. Cotton was grown in an alternating wide–narrow row pattern, with wide-row spacing of 80 cm, narrow-row spacing of 60 cm, and cotton plant spacing of 30 cm. From 8:00 to 9:00 every day, 2 DPA cotton bolls were taken back to the laboratory for in vitro ovule culture.

### 4.2. In Vitro Culture of Cotton Ovules

**Figure 4:**
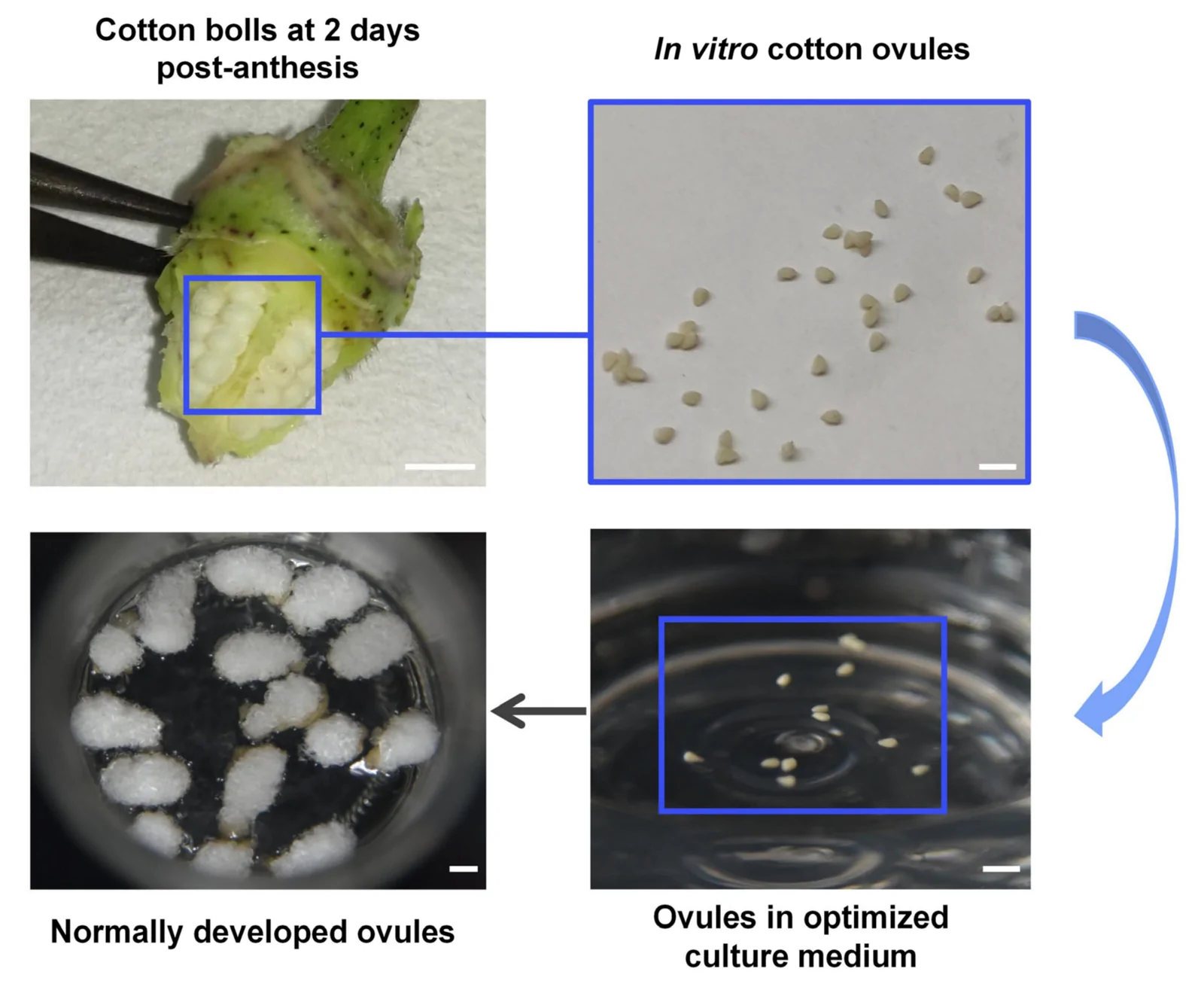
The experimental workflow for the in vitro culture of cotton ovules. This figure illustrates the key sequential steps, from ovule removal from the ovary to their transfer to the culture medium. It also presents a representative image of the ovules after successful fiber growth. Scale bars represent 3 mm.

The bracts, petals, stamens, pistils, and calyx were removed from the 2 DPA cotton bolls, and then the ovary was carefully opened. The ovaries were sterilized on an ultra-clean bench by first soaking them in 70% alcohol for 3–4 min (shaking continuously during the soaking process) and then in 0.1% mercuric chloride (HgCl_2_) for 8–9 min, followed by rinsing 3–4 times with sterilized distilled water. The ovaries were then placed on sterilized filter paper to drain. The ovules were removed with a scalpel, which had been burned with an alcohol lamp to disinfect it (note the sterile procedure). Next, 10–15 ovules were placed in each culture vial, and three replicates were set for each treatment. To record fiber development during ovule culture, fibers were observed and photographed using a camera; representative images are shown in Figure 4.

### 4.3. Medium Composition

We used a basal BT medium based on a previous study [11], supplemented with four different combinations of carbon sources: 0.05 M glucose, 0.05 M sucrose, and 0.02 M fructose; 0.1 M sucrose and 0.02 M fructose; 0.1 M glucose and 0.02 M fructose; 0.05 M glucose and 0.05 M sucrose. The pH of the medium was adjusted to 5.5 before high-temperature sterilization. After the culture medium was sterilized and cooled, 5.0 µM IAA and 5.0 µM GA_3_ were added. When exploring the effect of KT on the in vitro culture of ovules, we set two concentrations, 0.1 mg/L and 0.5 mg/L. When exploring the effect of Gln on the in vitro culture of ovules, we set two concentrations, 0.5 g/L and 1.0 g/L. In our experimental design, glucose, sucrose, and fructose were each tested as distinct carbon sources, either individually or in specific combinations. We clarified that glucose and fructose were fundamental monosaccharide carbohydrates. When glucose and fructose were included, they were added directly to the medium as purified monosaccharides, intended for direct uptake by the ovules.

### 4.4. Culture Observations

After the ovules were inoculated into the culture medium, they were incubated at (32 ± 1) °C in darkness. The growth status of the ovules and fibers was systematically evaluated (including quantitative measurements and qualitative observation) throughout the entire culture period.

Ovules were classified as “browned” if any visible browning was observed on the ovule surface; ovules with no visible browning were classified as “non-browned”. The browning rate was calculated using the following formula: Browning rate (%) = (Number of browned ovules/Total number of observed ovules) × 100. For each selected ovule, the fiber cluster growing on its outer surface was first gently flattened using forceps (to avoid fiber breakage and ensure accurate length reading, without separating individual fibers). Using a ruler with 0.1 mm accuracy, the maximum length of the fiber cluster (i.e., the distance from the base of the fiber cluster attached to the ovule surface to the farthest fiber tip in the cluster) was measured as the “overall fiber length” of the ovule. A qualitative visual assessment was conducted using a magnifying glass to evaluate the impact of glutamine on fiber fluffiness. The comparison focused on the looseness of fiber clusters, specifically distinguishing between their “tight, dense” and “loose, fluffy” appearances. The tight, dense appearance morphology was characterized by fiber clusters that were compact, had a low apparent volume, and grew close to the ovule surface with minimal space between individual fibers. The loose, fluffy appearance morphology was characterized by fiber clusters that were voluminous, had a high apparent volume, and grew with an expansive, open structure with abundant space between individual fibers.

For each of the repeated experiments, there were 15 ovules (with three repetitions for each experiment). No abnormalities (e.g., microbial contamination, ovule decay, or fiber degradation beyond oxidative browning) were noted during the 24-day incubation, and the medium was not changed during the culture period.

### 4.5. Image Acquisition and Processing

Images of cultured ovules were captured using a digital camera (Nikon D750). The typical settings used were an aperture of f/4, an ISO of 1600, and a shutter speed of 1/50 s. All photographs were taken under controlled lighting conditions to ensure consistent representation of the samples. Photographs were taken under natural diffused light to accurately represent the ovules’ color and overall appearance. Separately, direct flash lighting was used to highlight the density and morphological details of the fibers. All images were uniformly processed to adjust brightness and contrast, but color balance was maintained to prevent any misrepresentation of the results.

## 5. Conclusions

In this study, the in vitro culture system of upland cotton ovules was optimized by investigating the effects of carbon sources, kinetin, and glutamine on ovule growth and observed fiber morphology. Glucose and fructose in combination with their degradation products were the most suitable carbon sources for upland cotton ovule culture. In in vitro culture, 0.5 mg/L of KT can significantly promote the growth of ovule fibers. Gln appeared to promote a fuller, softer fiber appearance. Through the in vitro culture of ovules, the effects of plant growth regulators as well as other chemicals on cotton fiber cell growth can be studied, providing a more convenient and efficient method than functional verification in the field.

## Figures and Tables

**Table 1:** Optimized medium formulation.

The Composition of the Optimized Culture Medium	Concentration
BT medium	Inorganic–organic mixture [11]
Glucose (M)	0.05
Fructose (M)	0.02
IAA (µM)	5.0
GA_3_ (µM)	5.0
KT (mg/L)	0.5
Gln (g/L)	0.5

## Data Availability

The original contributions presented in this study are included in the article. Further inquiries can be directed to the corresponding authors.
